# Book Reviews

**Published:** 1897-11

**Authors:** 


### Book Reviews.

An Epitome of the History of Medicine. By Roswell Park, A. M., M. D., Professor of Surgery in the Medical Department of the University of Buffalo, etc. Illustrated with Portraits and other Engravings. One Volume, Royal Octavo, pages xiv–348. Extra Cloth. Beveled Edges, $2.00 net. The F. A. Davis Co., Publishers, 1914 and 1916 Cherry street, Philadelphia; 117 W. Forty-Second street, New York; 9 Lakeside Building, Chicago.

In the busy search after something new in the several provinces of medicine, one is apt to neglect to acquaint himself with the history of his art. It is a duty which every physician owes to the dignity of his calling to have an intelligent knowledge of its history, and it can be gained in no easier or more agreeable manner than by reading Dr. Roswell Park's book. The history is begun with an account of the medicine of the ancients and is brought through its various transitions up to the present. The most important eras and events are chronicled, and portraits of renown of all countries and times are freely interspersed throughout the volume. There are special chapters devoted to the history of antiseptics and anæsthesia; in the latter it is worthy of note that the claim of priority of discovery of ether as an anæsthetic is awarded to Crawford W. Long. The book is a neat volume of 341 pages and sells for the modest price of $2.00.

---

Twentieth Century Practice of Medicine. Volume XI. William Wood & Co.

Volume XI. includes in its contents diseases of the spinal cord, peripheral nerves and a Monograph on Pain. Like the other volumes of this series it has the rare virtue of being written up to the time it went to press. Diseases of the cerebro-spinal and sympathetic nerves are treated of in an elaborate lecture by James Hendric Lloyd. The practitioner will appreciate the ease with which he can refer to the spinal nerve palsies. The trophoneuroses including scleroderma, aeromegaly, etc., are contributed by Charles N. Mills and F. X. Dercum, and diseases of the cord by Bruns and Windschied, with the exception of tabes which has a section to itself by Mobius, and the combined system diseases by Adolph Strumpell.
The concluding Monograph on Pain by Lightner Witmer will probably be left unread by the busy physician who will therefore miss the most interesting and well-balanced article in the book.

REFERENCE BOOK OF PRACTICAL THERAPEUTICS. By various authors. Edited by Frank P. Foster, M. D., Editor of the New York Medical Journal and of Foster's Encyclopedic Medical Dictionary. In two volumes. Vol. II. D. Appleton & Co., New York, 1897.

The contributors to this volume, which begins with U and completes the subjeet with the X-ray, are S. I. Armstrong, E. B. Bronson, Brickner, Coley, Crandall, J. T. Eskridge, of Denver; M. L. Foster, A. G. Gerster, Griffin; Jewett, Lilienthal, R. H. Nevins, O'Malley, G. L. Peabody, F. Peterson, L. O. L. Potter, Chas. Rice, S. Solis, Cohen, Jas. T. Whitaker.

A vast amount of information is contained in the volume, yet the condensation is not so great as to interfere with a sufficiently full exposition of the subjects considered.

The work is elaborately indexed, containing not only a very full general index, but one of diseases and remedies.

The work is too large to commend itself as a text-book, but as a book of reference it has no equal.

---

ESSAYS ON SOCIAL TOPICS. By Lady Cook. Westminster. The Roxburghe Press, N. D. First, Second and Third Series. 1 Vol. Cloth.

There is a story familiar in the magazine world of a manuscript submitted by some noble lord and promptly rejected by the assistant editor on its merits. To his wonder it was accepted by the managing editor who told the assistant that there was nothing in it, but it was wonderful that a title could write at all. The anecdote comes vividly to mind on reading Lady Cook's Essays and it may account for their publication. A noted dramatic critic once said of a certain performance that it was a pity the actors should have to render such a play and that the play should be rendered by such players, and it is a pity that Her Ladyship's ideas should be expressed as they are and that she should deem it necessary to express such ideas.

The range of topics is wide but all turn on the social relations of the sexes. "Delicate subjects" are treated with unquestionable skill. Lady Cook avoids the brutal frankness of those of her sex who have of late years undertaken to enlighten the world on the master passion. She says nothing new nor does she clothe old facts and ideas with new force, but she has more sense of justice than most of her sisters. The chapters on "True Love," "Wrongs of Married Men" and "The Lost Rib" are perhaps the strongest in the book, and, after "The Heavenly Twins" and others of its type, it is refreshing to see a female author grant some virtue to man and allow that some women are imperfect.

We can hardly commend the work. To do any good on such subjects more force is needed than these essays possess, and unless good is done, harm follows such attempts. The ordinary fault of generalizing from insufficient data and a neglect of the undistributed middle, is seen and one must be a superficial thinker to get many new ideas from the volume.

C. M B.

AIROL PASTE IN THE TREATMENT OF WOUNDS.

Bruns (*Beitrage zur klinische Chirurgie*, xvii., 2; *Centralblatt fur Chirurgie*, July 17, 1897) recommends a paste of the following composition for covering sutured wounds:

℞. Airol ............... } each
Mucilage of gum arabic. } 10
Glycerin ............... } parts.
White bole ............ 20 parts.

M. The paste should be spread on evenly and covered with a thin layer of cotton and a slightly compressive bandage. It dries rapidly, adheres well, works powerfully as an antiseptic, is absolutely unirritating to the skin, and allows the serious secretion of the wound to pass through it.

Dr. G. T. Howland (*Jour. Cut. and G.-U. Diseases*) recommends the following non-irritating lubricant, which can be applied to all instruments which are to be used in the urethra.

℞. Pulv. white castile soap..... ℥i.

Aq. distil ................. ℥iii.

Mucil. chrondrus crispus... ℥iii.

Formalin (40 per cent)......mx.

Thymol ................. gr. v.

Ol. thyme..................mv.

Alcohol ...................mxv.

This is dispensed in two-ounce collapsible tubes, perpared by Fraser & Co., of New York.

